# A U.S. Partnership with India and Poland to Track Acute Chemical Releases to Serve Public Health

**DOI:** 10.3390/ijerph6092375

**Published:** 2009-09-03

**Authors:** Perri Zeitz Ruckart, Maureen Orr, Anna Pałaszewska-Tkacz, Aruna Dewan, Vikas Kapil

**Affiliations:** 1 Agency for Toxic Substances and Disease Registry, 4770 Buford Highway, MS F57, Atlanta, GA 30341, USA; E-Mail:morr@cdc.gov; 2 Scientific Information Department, Nofer Institute of Occupational Medicine, 8 Sw.Teresy Street, 91–348 Lodz, Poland; E-Mail:apalasz@imp.lodz.pl; 3 National Institute of Occupational Health (NIOH), Ahmedabad, Gujarat, 380016 India; E-Mail:dewanaruna@yahoo.com; 4 Centers for Disease Control and Prevention, National Center for Injury Prevention and Control, 4770 Buford Highway, MS F62, Atlanta, GA 30341, USA; E-Mail:vkapil@cdc.gov

**Keywords:** chemical surveillance, chemical release, public health

## Abstract

We describe a collaborative effort between the U.S., India, and Poland to track acute chemical releases during 2005–2007. In all three countries, fixed facility events were more common than transportation-related events; manufacturing and transportation/warehousing were the most frequently involved industries; and equipment failure and human error were the primary contributing factors. The most commonly released nonpetroleum substances were ammonia (India), carbon monoxide (U.S.) and mercury (Poland). More events in India (54%) resulted in victims compared with Poland (15%) and the U.S. (9%). The pilot program showed it is possible to successfully conduct international surveillance of acute hazardous substances releases with careful interpretation of the findings.

## 1. Introduction

International surveillance of acute chemical releases is a matter of security and public health protection [1]. To provide information for effective public health interventions to reduce morbidity and mortality, surveillance activities must be integrated with data analysis, effective, secure communication protocols, and methods that provide real-time, multi-directional information exchange.

In 1990 the U.S. Agency for Toxic Substances and Disease Registry (ATSDR) created an active, web-based surveillance system to capture the public health impact of acute releases of hazardous substances. When releases of toxic industrial chemicals (TICs) or biological agents occur, information is frequently lacking about both the short- and long-term effects of these releases on the exposed population [2]. Given the relative ease of global access to industrial chemicals and the current lack of resources, personnel, and expertise to respond to every incident, active surveillance is essential to identify TIC releases of potential public health significance [1,2]. Through program partners in participating state health departments in the U.S., ATSDR’s Hazardous Substances Emergency Events Surveillance (HSEES) actively collects information on acute releases of hazardous substances and associated public health outcomes (e.g., deaths, injuries, and evacuations).

In 2004, ATSDR began collaborating with India’s National Institute of Occupational Health (NIOH), one of the institutes of the Indian Council of Medical Research located in Gujarat, India and the Nofer Institute of Occupational Medicine (NIOM) in Lodz, Poland. The goal of this collaboration was to conduct pilot surveillance of acute chemical releases in these two countries using HSEES. This paper describes similarities and differences in the data among the U.S., India and Poland during 2005–2007.

## 2. Methods

The HSEES system has collected data on acute releases of hazardous substances and their associated injuries and evacuations in the U.S. for almost 20 years. In the United States, a HSEES event is defined as an uncontrolled and/or illegal acute release of any hazardous substance meeting specific pre-established criteria. Threatened releases of qualifying amounts of a hazardous substance are included if the threat leads to an evacuation or other action to protect the public health. The Petroleum Exclusion clause of the Comprehensive Environmental Response, Compensation, and Liability Act prohibits ATSDR from becoming involved with incidents where any form of petroleum was released if the material had not been refined to the point of becoming a specific chemical product such as pure xylene [3]. However, HSEES records information about petroleum if it was released with another qualifying substance. A variety of sources (e.g., records and oral reports of state environmental agencies, police and fire departments, and hospitals) are used in the U.S. to collect information about the acute hazardous chemical events. A victim is defined as a person experiencing at least one documented adverse health effect (such as respiratory irritation or chemical burns) that likely resulted from the event and occurred within 24 hours after the release. The HSEES system does not identify the immediate cause of the adverse health effect other than it happened during the course of the event.

Data are entered into a secure Web-based application that enables ATSDR to instantly access data except for company or personal identifiable information. Information collected for each event included data such as the location and industry involved in the event, chemicals released, number of victims, evacuations, and contributing factors for the event. For the analyses, the chemicals released are grouped into 16 categories: acids, ammonia, bases, chlorine, formulations, hetero-organics, hydrocarbons, mixture across categories, oxygenated organics, paints and dyes, pesticides, polychlorinated biphenyls (PCBs), polymers, volatile organic compounds (VOCs), other inorganic substances, and other substances. Mixture across categories consists of chemicals from different categories mixed prior to the incident. The category “other inorganic substances” comprises all inorganic substances, except acids, bases, ammonia, and chlorine, and includes chemicals such as nitrogen oxide and hydrogen sulfide. The “other” category consists of substances, such as asbestos, that could not be classified into any of the other 15 chemical categories.

International partners traveled to ATSDR offices in 2004 for orientation and training in HSEES. A site visit to a participating U.S. state was also made so the partners could experience a typical workday and investigation of HSEES events in a real-life setting. ATSDR scientists traveled to the NIOH offices located in Ahmedabad, Gujarat, India in 2004 and to the Nofer Institute in Lodz, Poland in 2005 to install the web-based HSEES system, attend meetings with stakeholders, and conduct additional training. Necessary equipment (computers, Internet connection, fax machine, printers, and photocopier) was procured and a full year of data entry began on January 1, 2005.

The HSEES data collected from the three countries for 2005–2007 were used in this analysis. In this time period 14 U.S. states participated in HSEES for the entire period: Colorado, Florida, Iowa, Louisiana, Michigan, Minnesota, New Jersey, New York, North Carolina, Oregon, Texas, Utah, Washington, and Wisconsin. The estimated population for these 14 states in 2007 was 125.075 million [4]. The state of Missouri also participated only in 2005. Major industries for these 14 states include construction; retail trade; professional, scientific, and technical services; and health care and social assistance which is similar to the U.S. as a whole [4].

For India, the case definition was modified to meet the needs of the in-country international partner. In India, releases of petroleum were included if the amount released was greater than 1,000 liters. Mass poisonings were also included. The case definition was not modified in Poland. In 2006 Poland dropped reporting of releases of mercury from private households because these spills, primarily associated with broken mercury thermometers, were thought to represent minimal amounts of mercury.

In India, surveillance for the pilot project was limited to Gujarat state where NIOH is located. Gujarat is a large state in Western India with a population of approximately 55.808 million in 2007 [5]. In a recent business census, Gujarat was found to be home to nearly 34,000 factories and industrial facilities (Personal communication, Directorate of Industrial Safety and Health, India). Major industries include oil and petroleum products, refineries, mining, and heavy manufacturing operations producing steel and aluminum. Over 750,000 people in Gujarat are estimated to be employed in these industrial facilities. Gujarat also has a large agricultural sector [6]. The primary notification source for events was the media, although reporting mechanisms had been established with the fire brigade and police. Regional data collectors were responsible for data collection, and data were entered by a central data entry person under the oversight of the Principal Investigator.

In Poland, surveillance included the entire country. Poland is the ninth largest country in Europe (312,679 km^2^) with a population of approximately 38.115 million in 2007 [7]. It is about the size of New Mexico. Major industries include coal mining and processing, power production, iron and steel sectors, machinery, electrical machinery and electronics production, cars and shipbuilding, textiles, and chemical production. Poland also has a large number of private agriculture farms employing about 16% of the work force [8,9]. The primary notification source for events was the fire department headquarters which collects reports about every accident cleaned up by fire fighters. Data were electronically transferred to Polish HSEES investigators on a monthly basis. A summary of the events in the U.S., India, and Poland are presented, as well as descriptive statistics from analyses comparing the most common industries in each country.

## 3. Results

During 2005–2007, 23,818 events or 190.43 events per million persons were captured by the U.S. HSEES system; 491 events or 8.798 events per million persons were captured by the India HSEES system; and 567 events or 14.876 events per million persons were captured by the Poland HSEES system. In all three countries, fixed facility events were more common than transportation-related events; however, Poland had a higher percentage of transportation-related events than the U.S. or India (37% vs. 30% and 29%, respectively). Additionally, more transportation events in Poland involved transport by rail compared with the U.S. or India (24% vs. 9% and 7%, respectively). Equipment failure and human error were the primary contributing factors for most events in all three countries: in India and Poland, human error accounted for more releases than equipment failure (Table 1a).

Improper filling/loading/packing was the most common secondary contributing factor in the U.S. and Poland while fire was most common in India (Table 1b).

Manufacturing and transportation/warehousing were the most frequent industries involved in events in all three countries accounting for 65% of the events in the U.S., 44% of the events in India, and 56% of the events in Poland (Table 2). Transportation/warehousing-related events occurred more frequently than manufacturing events in Poland.

The most commonly released substance categories were VOCs (19%) and other inorganic substances (18%) in the U.S., other (25%) and pesticides (16%) in India, and other inorganic substances (28%) and acids (21%) in Poland (Table 3). In the U.S., the three most frequently released individual substances were carbon monoxide (6%), ammonia (5%), and sulfur dioxide (4%). In Poland, mercury (15%), ammonia (9%), and hydrochloric acid (8%) were the most frequently released. In India, liquefied petroleum gas (17%), methane (4%), and natural gas (4%) were the most frequently released. Besides petroleum in India, the three most frequently released individual substances were ammonia (3%), monocrotophos (3%), and imidacloprid (2%). Approximately 62% of the mercury events in Poland occurred in private residences and almost all were due to human error involving broken thermometers. Poland collected information on mercury releases in private households in 2005, but excluded these events in 2006 and 2007 because they felt it skewed the data and the amount of mercury released was minimal. Twenty percent of all events in Poland resulted in an evacuation compared with 6% in the U.S. and 5% in India; the median number of people evacuated per event was 30, 50, and 20 people, respectively.

While a greater percent of incidents in India (54%) and Poland (15%) had victims compared with the U.S. (9%), there were fewer victims per million population in India (13.31) and Poland (11.68) compared with the U.S. (52.46). Victims were more likely to be admitted to a hospital in India and Poland compared with the U.S. (Table 4). While there were more victims in the U.S., fatalities were higher in India (281 deaths, 5.0 deaths per million persons) compared with the U.S. (207 deaths, 1.66 deaths per million persons) and Poland (4 deaths, 0.10 deaths per million persons).

Employees were the most frequently injured population group in the U.S. and India while students were the most frequently injured group in Poland. Five events involving 210 student-victims in Poland were due to the intentional release of pepper spray at schools.

Below is a more in-depth comparative analysis of the most prevalent industry categories in the three countries: Manufacturing; Transportation/Warehousing; Agriculture, Forestry, Fishing, and Hunting; and Other Services. These industry segments represent the three industries most associated with releases in India and Poland.

### 3.1. Manufacturing

Ammonia was the most frequently released individual chemical in manufacturing events in all three countries. Equipment failure and human error were the most common primary factors for these events, but human error accounted for more events in India and Poland than in the U.S. (38%, 31%, and 13%, respectively). The majority of the victims in manufacturing events in all three countries were employees (70% in the U.S., 89% in India, and 94% in Poland). Most victims of these events in India and Poland were admitted to a hospital while most victims of these events in the U.S. were treated at a hospital and released. Respiratory irritation (32%) was the most commonly reported injury or symptom in the U.S. compared with chemical burns (46%) in India, and dizziness/CNS effects (29%) in Poland. Over a third of the victims in manufacturing events in India died, compared with 2% in the U.S. and no fatalities in Poland.

### 3.2. Transportation/Warehousing

Paint or coating not otherwise specified was the most frequently released individual chemical in transportation/warehousing events in the U.S., while ammonia was the chemical most frequently released in Poland and liquefied petroleum gas was the most frequently released chemical in India and hydrochloric acid was the most frequently released non-petroleum chemical in India. The majority of the victims in the U.S. and Poland were employees (57% and 70%, respectively), while the majority of the victims in India were members of the general public (68%). Most victims of transportation/warehousing events in the U.S. were treated at a hospital and released, while most victims of these events were admitted to a hospital in India or treated at the scene in Poland. About a third of the victims in transportation/warehousing events in India died compared with 12% in the U.S. and 9% in Poland. Chemical burns were the most common injury among transportation/warehousing victims in India compared with trauma in both the U.S. and Poland.

### 3.3 Agriculture, Forestry, Fishing, and Hunting

Most of the agriculture-related events in India were due to human error (primary factor) and involved an overspray (secondary factor), while equipment failure and vehicle derailment were the most frequent primary and secondary factors in the U.S. and human error and unauthorized dumping were the most frequent primary and secondary factors in Poland. Ammonia (36%) was the most commonly released individual substance in these events in the U.S. and monocrotophos (16%) and imidacloprid and organophosphate (13% each) were the most frequently released individual substances in India. Mercury (33%) was the most frequently released substance in Poland; however, there were only six agricultural-related events in Poland. Almost all of the victims of these events in India were employees (99%) compared with 62% of the victims categorized as employees in the U.S.; there were no victims from events in this industry in Poland. Respiratory irritation (20%) was the most commonly reported injury or symptom in the U.S. and dizziness/CNS symptoms (51%) was the most commonly reported injury or symptom in India. One percent of the victims in agricultural-related events in the U.S. died compared with 86% in India. None of the employee- or responder-victims in India were reported to have worn personal protective equipment (PPE) while 12% of the employee- and responder-victims in the U.S. reported wearing PPE.

### 3.4 Other Services

Private households were the most common sub-type for events in the other services industry in the U.S., India, and Poland, accounting for 87%, 59%, and 98% of events in this group, respectively. About 25% of the other services events in the U.S. were related to illicit drug production (e.g., methamphetamine), while there were no events in India or Poland due to this contributing factor. Carbon monoxide (10%) was the most commonly released individual substance in other services events in the U.S., compared with liquefied petroleum gas (63%) in India which is primarily used for cooking (the top three individual substances released in India were all petroleum-related), and mercury (52%) in Poland. Most victims were members of the general public (80% in the U.S., 70% in India, and 68% in Poland). Respiratory irritation was the most commonly reported injury or symptom in the U.S. (25%) and Poland (41%), while chemical burns (55%) were the most frequent in India. Almost two-thirds of the victims in these events in India were admitted to a hospital, compared with 17% in the U.S., and 32% in Poland. There were no deaths from these events in Poland, compared with 5% in the U.S., and 27% in India.

## 4. Discussion

Fatalities from acute hazardous substance releases were much higher in India than in the U.S. and Poland. One reason for this may be that fire was the most frequent secondary contributing factor in events in India: fires are more likely to lead to fatalities than other types of secondary factors such as improper filling/loading/packing which was the most frequent secondary factor in the U.S. and Poland. However, Poland may not have captured secondary factors for all events. Another reason for the higher fatalities is that India included petroleum releases, which are likely to result in fires and explosions. India had the fewest reported number of incidents and victims per capita compared with the U.S. and Poland. This difference may be because the primary notification source for events in India was the media, and the media are more likely to report on high profile events with serious consequences including more serious injuries and fatalities.

Poland had a higher percentage of transportation-related events than the U.S. or India, and more transportation events in Poland involved transport by rail. Poland is a transit country for rail transport between east and west Europe. Rail events in Poland were frequently due to valve failures in stationary tankers. Age and quality of the tankers may be the reason for higher number of spills in this sector.

While the majority of the victims in manufacturing events in all three countries were employees, the percentage of employee-victims was higher in Poland and India than in the U.S. This may be because the U.S. has laws to protect the safety and health of people at work that include helping people understand the potential dangers of the hazardous chemicals they work with and providing education and training to workers about chemical hazards in the workplace [10].

Indian data suggests a higher mortality rate compared to Poland and the United States. In addition, a far higher proportion of Indian victims were admitted to a hospital and very few victims were treated at the scene. Although some of these differences may be attributable to injury severity, a number of other critical health system differences may have also impacted injury morbidity and mortality due to hazardous substance releases in India.

Emergency medical services (EMS) in India are in early stages of development and timely access to such services is generally available only in a few major metropolitan centers [11]. When available, formal pre-hospital EMS transport services are often poorly equipped and lacking trained pre-hospital care staff. Therefore, much pre-hospital care and transport services are provided through informal means and delivered by untrained bystanders [11]. After arrival, many receiving health care institutions lack the appropriate staff and resources to treat critically ill or injured patients [12].

Like other low and middle income countries, access to specialized care for victims exposed to hazardous substances is very limited. The World Health Organization’s (WHO) International Programme on Chemical Safety has identified only four functioning poison centers in all of India [13]. In addition to issues related to availability of appropriate staff, resources and facilities, and transportation issues, significant financial barriers to health care access also exist. For critical health care needs in India, public health care services are limited and many victims lack resources to pay for these services in the private sector. Additionally, financially viable health insurance options are largely absent [14].

The most frequently released chemicals in agriculture-related events in India (monocrotophos, imidacloprid, and organophosphate) are all neuro-toxic insecticides [15] and none of the victims wore PPE which may account for the high percentage of deaths in India. Monocrotophos was banned in the U.S. because of its acute toxicity to birds and humans [16].

The most frequently released substance in Poland, mercury, was mostly due to collecting data on broken thermometers in private households in 2005 which was discontinued in 2006. Moreover in April 2009 in Poland, medical equipment with mercury (including thermometers) was restricted from entering the market due to European Union legislation. The chemical category Acids was more frequently released in Poland than in the U.S. or India. This may be because companies engaged in fertilizer production, where acids are a popular substrate, represent a large portion of the Polish manufacturing sector.

## 5. Conclusions

The pilot program shows that HSEES can be used to successfully conduct international surveillance of acute hazardous substances releases. There are similarities in acute hazardous substances releases among the three countries, but because of differences in reporting methods, types of industries, culture, and degree of industrialization, direct comparisons among the countries should be carefully interpreted. These analyses illustrate the importance of thoroughly describing the methods and criteria involved in an international surveillance program in order to more accurately understand comparisons. The U.S. plans to continue a state-based surveillance program as part of a larger national surveillance program for toxic substance incidents and hopes to collaborate with other countries in the future.

## Disclaimer

The findings and conclusions in this report are those of the authors and do not necessarily represent the views of the Agency for Toxic Substances and Disease Registry.

## Figures and Tables

**Table 1a.** Primary factors involved in Hazardous Substances Emergency Events: U.S., India, and Poland, 2005–2007.

**Primary factor**	**U.S.**		**India**		**Poland**	

	**No.**	**%**	**No.**	**%**	**No.**	**%**

Bad weather	621	2.6	3	0.6	3	0.5
Equipment failure	11437	48.0	198	40.3	236	41.6
Human error	8766	36.8	233	47.5	287	50.6
Illegal act	718	3.0	8	1.6	1	0.2
Intentional	1983	8.3	0	0.0	35	6.2
Other	77	0.3	3	0.6	5	0.9
Unknown	216	0.9	46	9.4	0	0.0

Total*	23818	99.9	491	100.0	567	100.1

* percentages may not total 100% due to rounding.

**Table 1b.** Secondary factors involved in Hazardous Substances Emergency Events: U.S., India, and Poland, 2005–2007.

**Secondary factor**	**U.S.**		**India**		**Poland**	

	**No.**	**%**	**No.**	**%**	**No.**	**%**

Equipment failure	1010	4.2	29	5.9	0	0.0
Explosion	137	0.6	22	4.5	2	0.4
Fire	675	2.8	112	22.8	0	0.0
Forklift puncture	731	3.1	0	0.0	0	0.0
Human error	202	0.8	42	8.6	0	0.0
Illicit drug production	597	2.5	0	0.0	0	0.0
Improper filling/loading/packing	3297	13.8	9	1.8	44	7.8
Improper mixing	236	1.0	1	0.2	1	0.2
Loadshift	197	0.8	0	0.0	0	0.0
Overspray/misapplication	231	1.0	21	4.3	7	1.2
Performing maintenance	1266	5.3	2	0.4	1	0.2
Power failure	522	2.2	5	1.0	0	0.0
System/process upset	1484	6.2	4	0.8	14	2.5
System start up/shutdown	1340	5.6	0	0.0	0	0.0
Unauthorized dumping	752	3.2	2	0.4	27	4.8
Vehicle collision	391	1.6	10	2.0	21	3.7
Vehicle derailment/rollover	510	2.1	13	2.6	0	0.0
Other	257	1.1	0	0.0	2	0.4
No secondary factor	9865	41.4	161	32.8	448	79.0
Unknown	118	0.5	58	11.8	0	0.0

Total*	23818	99.8	491	99.9	567	100.2

* percentages may not total 100% due to rounding.

**Table 2.** Industries involved in Hazardous Substances Emergency Events: U.S., India, and Poland, 2005–2007.

**Industry category**	**U.S.**		**India**		**Poland**	

	**No.**	**%**	**No.**	**%**	**No.**	**%**

Accommodation and food services	143	0.6	14	2.9	7	1.2
Agriculture, Forestry, Fishing, and Hunting	458	1.9	81	16.5	6	1.1
Arts, Entertainment, and Recreation	147	0.6	1	0.2	9	1.6
Construction	148	0.6	0	0.0	6	1.1
Educational services	384	1.6	1	0.2	48	8.5
Health care and social assistance	226	0.9	2	0.4	10	1.8
Manufacturing	8928	37.4	133	27.1	108	19.0
Mining	318	1.3	4	0.8	3	0.6
Other services	1115	4.7	71	14.5	106	18.7
Other*	45	0.2	0.0	0.0	1	0.2
Professional services	87	0.4	0	0.0	5	0.9
Public administration	248	1.0	0	0.0	3	0.5
Real estate	284	1.2	0	0.0	1	0.2
Retail trade	348	1.5	23	4.7	5	0.9
Transportation/Warehousing	6582	27.6	84	17.1	212	37.4
Utilities	1149	4.8	37	7.5	10	1.8
Waste management and remediation	393	1.7	1	0.2	24	4.2
Wholesale trade	1389	5.8	5	1.0	1	0.2
Not an industry	1126	4.7	26	5.3	0	0.0
Not identified	300	1.3	8	1.6	2	0.4

Total†	23818	99.8	491	100.0	567	100.3

* includes Finance, Information, and Management of companies;

† percentages may not total 100% due to rounding.

**Table 3.** Substance categories involved in Hazardous Substances Emergency Events: U.S., India, and Poland, 2005–2007.

**Substance category**	**U.S.**		**India**		**Poland**	

	**No.**	**%**	**No.**	**%**	**No.**	**%**

Acids	2706	8.8	40	7.8	148	21.4
Ammonia	1522	4.9	14	2.7	65	9.4
Bases	1478	4.8	6	1.2	30	4.3
Chlorine	883	2.9	6	1.2	28	4.0
Formulations	36	0.1	0	0.0	0	0.0
Hetero-organics	178	0.6	23	4.5	3	0.4
Hydrocarbons	402	1.3	23	4.5	10	1.4
Mixture across categories	3316	10.8	6	1.2	17	2.5
Other	1637	5.3	127	24.6	33	4.8
Other inorganic substances	5686	18.4	25	4.8	191	27.6
Oxy-organics	2933	9.5	19	3.7	59	8.5
Paints and dyes	1760	5.7	3	0.6	9	1.3
Pesticides	1270	4.1	80	15.5	22	3.2
Polychlorinated biphenyls	284	0.9	0	0.0	0	0.0
Polymers	809	2.6	9	1.7	12	1.7
Volatile organic compounds	5912	19.2	79	15.3	59	8.5
Indeterminate	29	0.1	56	10.9	6	0.9

Total*	30841	100.0	516	100.2	692	99.9

* percentages may not total 100% due to rounding.

**Table 4.** Disposition of victims in Hazardous Substances Emergency Events: U.S., India, and Poland, 2005–2007.

**Disposition**	**U.S.**		**India**		**Poland**	
	**No.**	**per 1,000,000 persons**	**No.**	**per 1,000,000 persons**	**No.**	**per 1,000,000 persons**
Admitted to a hospital	696	5.56	360	6.45	183	4.80
Death	207	1.66	281	5.04	4	0.10
Injuries reported by an official	283	2.26	8	0.14	0	0.00
Observed at hospital	164	1.31	0	0.00	128	3.36
Seen by private physician	150	1.20	0	0.00	0	0.00
Treated at a hospital (not admitted)	3284	26.26	48	0.86	75	1.97
Treated on scene or mass casualty unit	1410	11.27	46	0.82	51	1.34
Unknown	367	2.93	1	0.02	4	0.10
Total	6561	52.46	744	13.33	445	11.68
